# Supplementary material for: Encapsulating TGF-β1 Inhibitory Peptides P17 and P144 as a Promising Strategy to Facilitate Their Dissolution and to Improve Their Functionalization
Source: Pharmaceutics. 2020 May 2;12(5):421. doi: 10.3390/pharmaceutics12050421 (PMC7284799; doi:10.3390/pharmaceutics12050421)
Supplement: Supplementary file 1 [file pharmaceutics-12-00421-s001.pdf]

pharmaceutics MDPI

Supplementary Materials: Encapsulating TGF-β1 Inhibitory Peptides P17 and P144 as a Promising Strategy to Facilitate their Dissolution and to Improve their Functionalization

Nemany A. N. Hanafy, Isabel Fabregat, Stefano Leporatti and Maged El Kemary

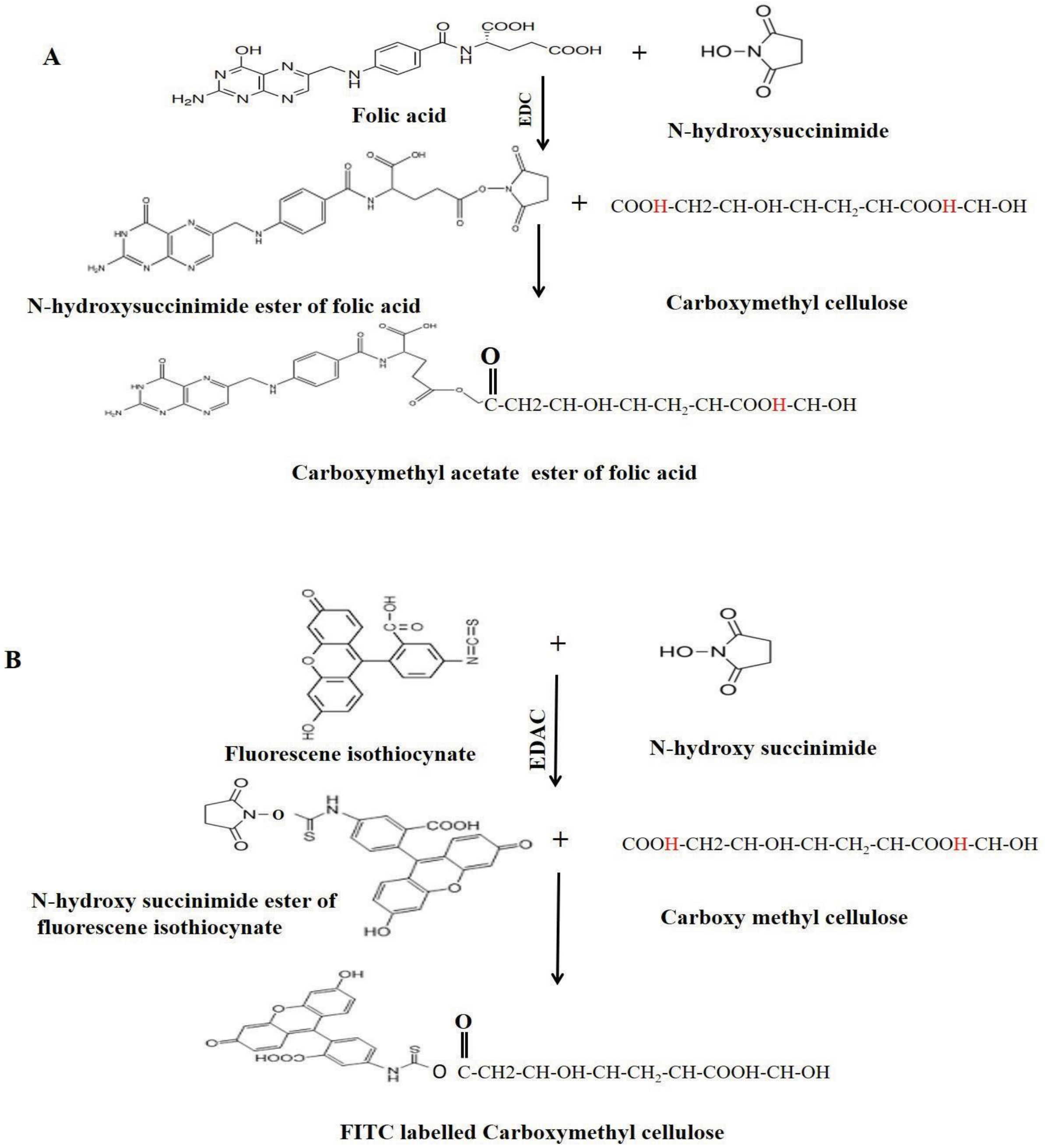

Scheme S1: (**A**): Folic Acid conjugated carboxymethyl cellulose. (**B**) Labelling carboxymethyl cellulose with FITC.

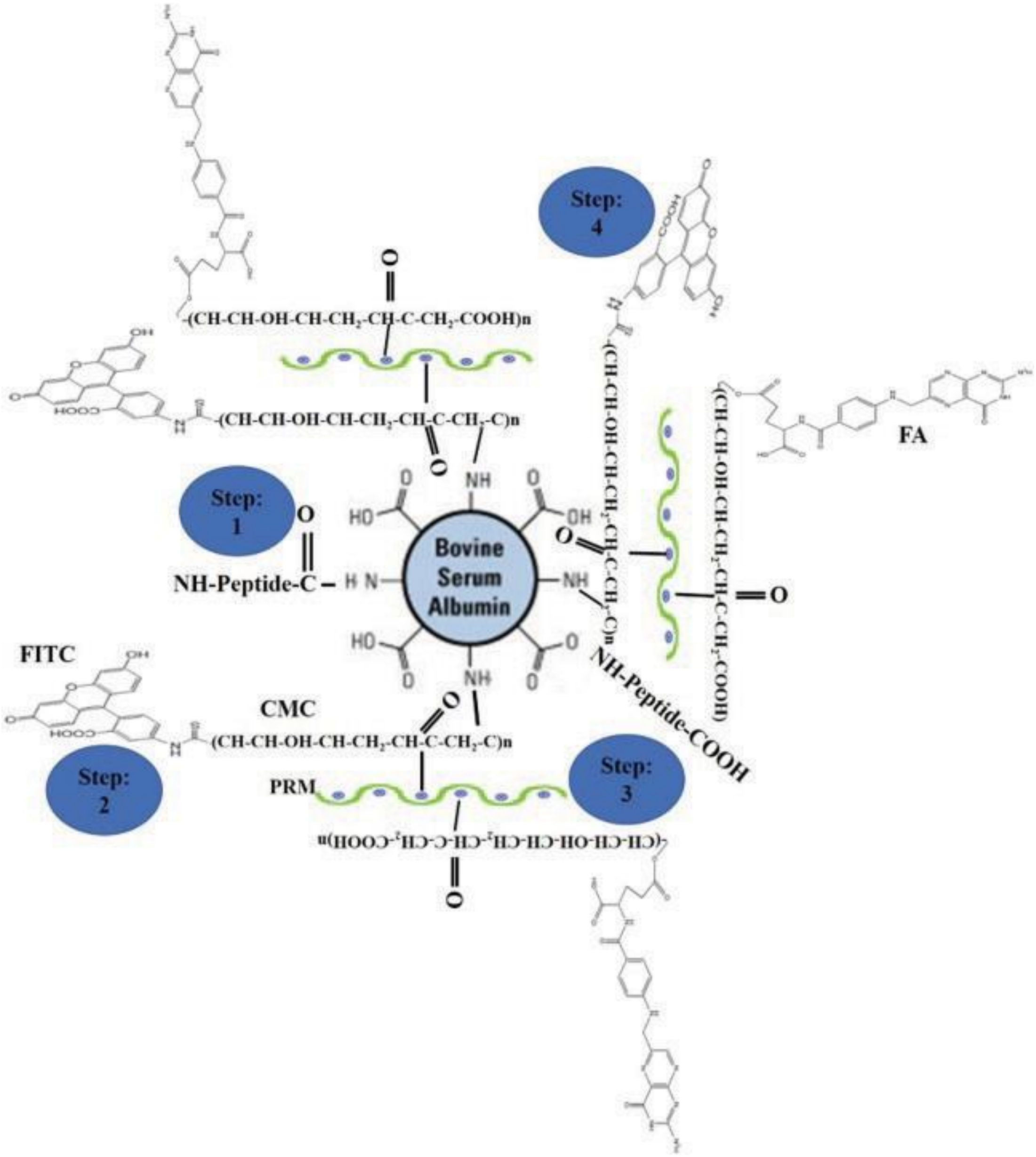

Scheme S2: Fabrication of protein mucoadhesive targeted therapy. Step 1: interaction of BSA and peptide. Step 2: protein peptide complex coated by CMC alone or with FITC-CMC in case of free capsules. Step 3: addition of protamine (PRM). Step 4: functionalization of all the structure with folic acid conjugated carboxymethyl cellulose.

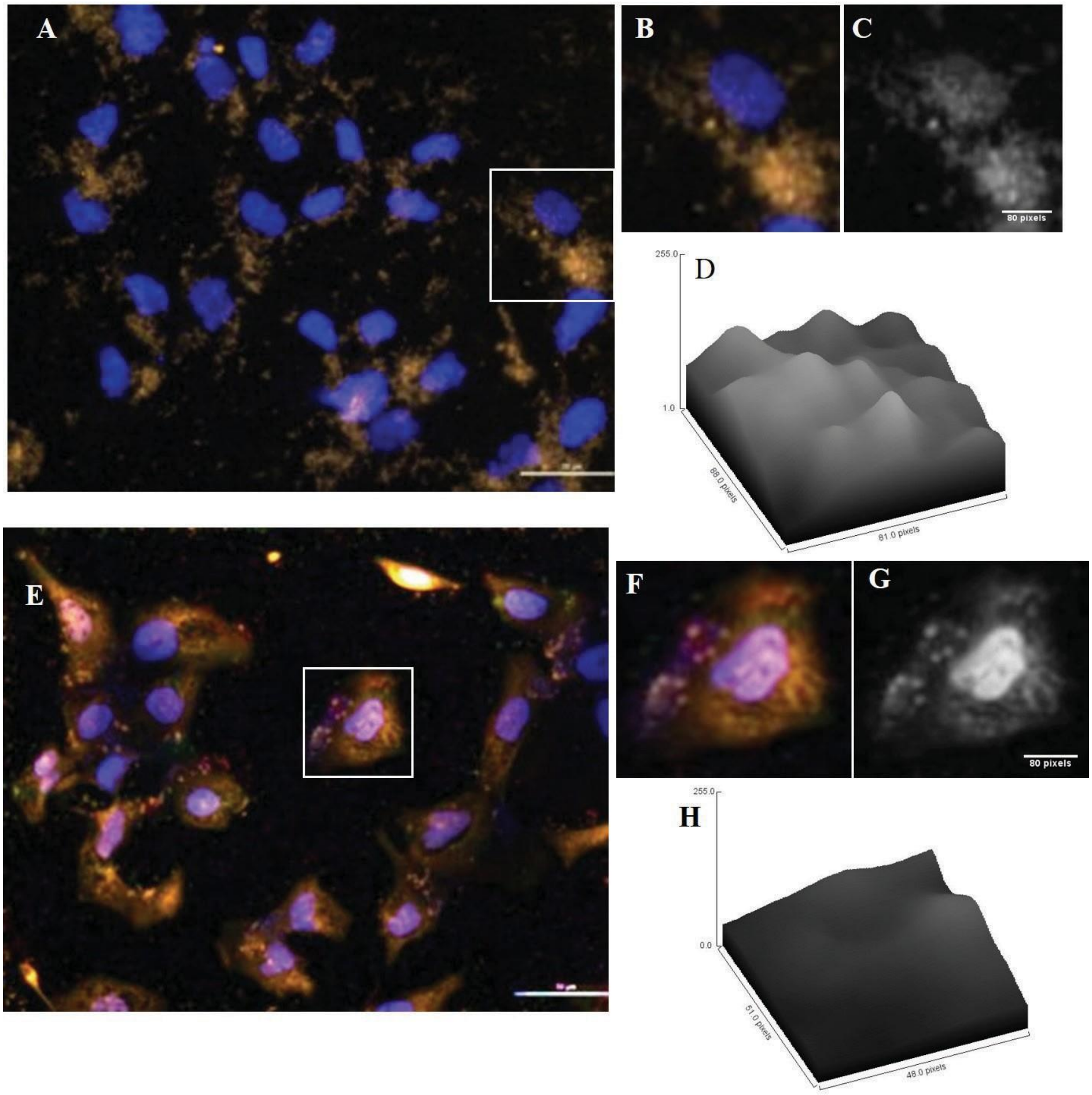

Figure S1. Fluorescence microscopy images for labelled P144. (**A**) Not encapsulated labelled p144. (**B**) Magnification of few cells; (**C**) Image J analysis " Grayscale image"; (**D**) Image J analysis " surface plot"; (**E**) Encapsulated labelled P144; (**F**) Magnification of few cells . (**G**) Image J analysis " Grayscale image"; (**H**) Image J analysis " surface plot".
